# Modeling Dynamic Human Behavioral Changes in Animal Disease Models: Challenges and Opportunities for Addressing Bias

**DOI:** 10.3389/fvets.2018.00137

**Published:** 2018-06-21

**Authors:** Arata Hidano, Gareth Enticott, Robert M. Christley, M. Carolyn Gates

**Affiliations:** ^1^EpiCentre, School of Veterinary Science, Massey University, Palmerston North, New Zealand; ^2^Cardiff School of Geography and Planning, Cardiff University, Cardiff, United Kingdom; ^3^Department of Epidemiology and Population Health, Institute of Infection and Global Health, University of Liverpool, Neston, United Kingdom; ^4^Institute of Veterinary Science, University of Liverpool, Neston, United Kingdom

**Keywords:** feedback loop, human behavior, behavioral change, infectious disease model, livestock disease, network analysis, qualitative study, psychological and economic model

## Abstract

Over the past several decades, infectious disease modeling has become an essential tool for creating counterfactual scenarios that allow the effectiveness of different disease control policies to be evaluated prior to implementation in the real world. For livestock diseases, these models have become increasingly sophisticated as researchers have gained access to rich national livestock traceability databases, which enables inclusion of explicit spatial and temporal patterns in animal movements through network-based approaches. However, there are still many limitations in how we currently model animal disease dynamics. Critical among these is that many models make the assumption that human behaviors remain constant over time. As many studies have shown, livestock owners change their behaviors around trading, on-farm biosecurity, and disease management in response to complex factors such as increased awareness of disease risks, pressure to conform with social expectations, and the direct imposition of new national animal health regulations; all of which may significantly influence how a disease spreads within and between farms. Failing to account for these dynamics may produce a substantial layer of bias in infectious disease models, yet surprisingly little is currently known about the effects on model inferences. Here, we review the growing evidence on why these assumptions matter. We summarize the current knowledge about farmers' behavioral change in on-farm biosecurity and livestock trading practices and highlight the knowledge gaps that prohibit these behavioral changes from being incorporated into disease modeling frameworks. We suggest this knowledge gap can be filled only by more empirical longitudinal studies on farmers' behavioral change as well as theoretical modeling studies that can help to identify human behavioral changes that are important in disease transmission dynamics. Moreover, we contend it is time to shift our research approach: from modeling a single disease to modeling interactions between multiple diseases and from modeling a single farmer behavior to modeling interdependencies between multiple behaviors. In order to solve these challenges, there is a strong need for interdisciplinary collaboration across a wide range of fields including animal health, epidemiology, sociology, and animal welfare.

## Introduction

Since the seminal application of infectious disease models to the 2001 foot-and-mouth disease (FMD) outbreak in the United Kingdom (UK), the number of published modeling studies for livestock diseases has increased dramatically (1, 2). These models can be useful tools for evaluating the efficacy of different disease control strategies especially in situations where it may not be ethically justifiable or where it may be too time consuming and expensive to perform research studies in the real world. Livestock infectious disease models are generally built as follows. First, the population demographic structure and various disease transmission pathways are identified based on existing knowledge about the disease system. The within-farm and between-farm disease transmission dynamics are then modeled, if necessary, by defining contact patterns over time and relevant parameters to describe the likelihood of transmission occurring through those contacts. Once the baseline disease dynamic model has been developed and validated, the effectiveness of various control strategies may then be evaluated by imposing modifications on the system.

Model structure has also become increasingly complex, evolving from simple compartmental models where each farm does not have an identity all the way to sophisticated individual-based models where the population is divided into a number of subpopulations that are typically spatially separated and each animal is individually identified and traced throughout the simulation. With the increasing availability of national livestock movement records, researchers can also explicitly replicate livestock movement patterns that occurred in the past to realistically simulate how disease spreads along with movements. With more thorough sensitivity analyses being performed, it is also possible to test the influence of recognized model assumptions and limitations on the final control recommendations (3). Using these tools and methodologies, infectious disease dynamics can be studied with an unprecedentedly high resolution. However, most models still assume constant human behavior meaning that the patterns of within- and between-farm contacts as well as the risk of transmission through the contact remain constant in the models even though farmers in the real world may need to adapt their behaviors to deal with the disease and the control strategy imposed upon them (4–8).

Both empirical and theoretical studies for human diseases clearly show that behavior can have a substantial impact on disease epidemiology. For instance, in the recent Ebola outbreaks in Africa, it was found that traditional funerals in West Africa that involve family members washing the corpse contributed significantly to the number of secondary infections with Ebola virus (9, 10). Vaccine refusal or vaccine hesitancy can also occur in a spatially clustered manner for various reasons, including shared views within a community or poor financial status in an area, and this can substantially increase a risk of infection in the geographical area (11). Not only that, it is known that humans change behaviors in response to various factors including disease occurrence, increased awareness toward a disease risk, social norm, and the perceived efficacy of a disease control strategy (6, 7). In the same example of the Ebola outbreak, Abramowitz et al. showed how the local community's beliefs about the source and transmission of Ebola changed during the outbreak period, which subsequently changed how people implemented infection prevention and control measures to protect their own health status (12).

Similar findings have been observed with animal diseases. For example, a qualitative study on horse owners' perception toward Hendra virus revealed that some people share a belief that vaccinations may lead to adverse reactions in horses such as decreased performance, abortion, and death (13). Some owners also believed that the vaccine was not tested rigorously enough due to it being developed in in a rush, which has been identified in other human behavioral studies as yet another reason why people may fail to adopt vaccinations (14). Regarding other dynamic human behaviors, empirical observations suggest that live bird market closures in response to avian influenza outbreaks may induce an undesirable behavioral change in poultry owners, such as increasing the frequency of movements of high-risk animals to avoid culls or performing illegal trading through an underground markets (15, 16) both of which contribute to further disease spread (17). Other studies have similarly reported that movement restrictions can result in infected livestock being sold from an area where disease outbreak occurs (18, 19). Emergence of undesirable behaviors has been also observed amongst some UK farmers, who performed illegal badger culling to control bovine tuberculosis (bTB) because they did not trust the government and hence its legislation (20).

Two sets of behaviors in particular have been identified as being highly influential for human disease spread; one as behaviors related to determining the mixing or contact patterns between hosts and the other as behaviors related to disease prevention and control (8). Since livestock populations are managed by humans, it is only natural that similar behavioral factors can influence the epidemiology of livestock diseases. In the context of livestock diseases, these behaviors translate into livestock contact patterns and the biosecurity practices farmers take to prevent disease from spreading through these contacts. The complex interrelationship between disease spread and dynamic human behavior needs to be accounted for in disease simulation models to minimize potential bias in inferences (21).

**Figure 1:**
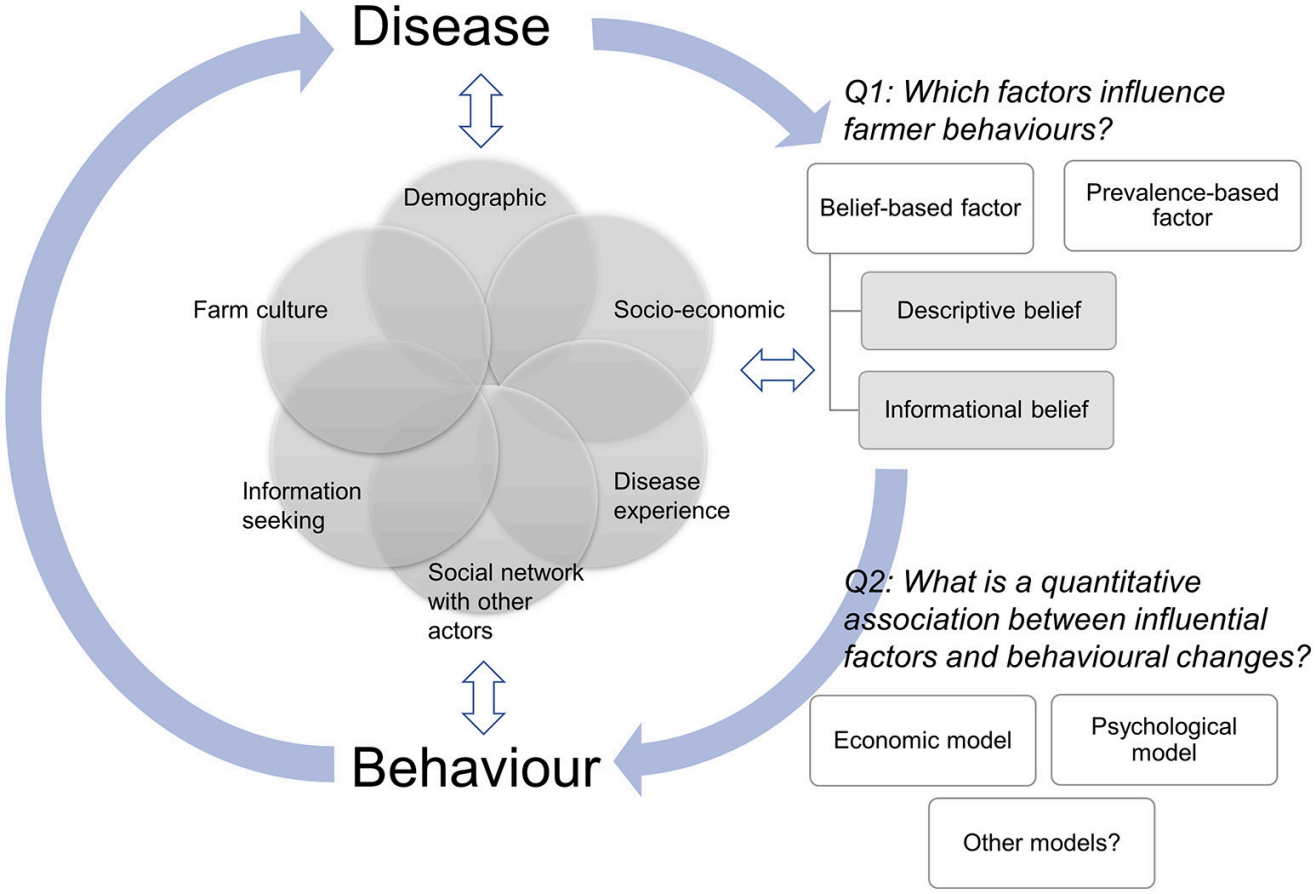
Schematic diagram of the feedback loop between disease and behavior. Disease influences farmer behaviors through prevalence-based and belief-based factors, which are influenced by various farms' and farmers' characteristics (e.g., demographic factors, socio-economic status, and social network with other actors). Behavior in turn changes disease dynamics. Farms' and farmers' characteristics influence both disease and behavior, which also influence farms' and farmers' characteristics. We highlighted two key areas (Q1 and Q2) that need further studies in order to accurately capture the inter-relationships between disease and dynamic human behaviors.

This logically leads to the questions of how much detail of dynamic human behavioral change do we actually need to capture to make valid modeling inferences and how should we best model dynamic human behavioral changes? Answering these questions will require an understanding of (1) the disease-related factors that are most likely to cause behavioral change including epidemiological factors (e.g., knowledge of disease prevalence, incidence, and mortality rates) as well as other broader psychological and social factors (e.g., farmers' perception of disease risk and the disease experience of neighboring farmers) and how they change human behaviors, and (2) what methods we can use to quantitatively model the association between changes in these disease-related factors and changes in a behavior. Fortunately, the rapidly growing literature on farmers' behaviors provides greater knowledge on “what” may affect their behaviors. However, we still lack a solid understanding of “how” these factors operate and interact to deliver a dynamic human behavior, as represented in Figure 1.

In this review paper, we first summarize the existing literature around the disease-related factors that are responsible for changing farmers' behaviors (Section Disease-Related Factors Relevant to Farmers' Dynamic Behavioral Change). We then discuss different methods that currently exist for building dynamic human behavioral change into disease simulation models (Section Methods for Modeling Dynamic Human Behavioral Changes). This paper concludes with a discussion of challenges and opportunities for future research (Section Discussion).

## Disease-related factors relevant to farmers' dynamic behavioral change

Funk et al. proposed a system for classifying the disease-related factors that can lead to human behavioral change based on both the source of information (global or local) and the type of information (prevalence-based vs. belief-based) that individuals routinely use to make personal health decisions (7). Global information refers to disease information that is widely available in the public domain through national television, newspapers, magazines, and government information services. Local information refers to disease information that is only circulated amongst close social neighbors such as discussions between neighboring farmers or local farming groups. It is important to distinguish between these two sources because local knowledge may lead to significant local and regional variation in human behavior (e.g., clustered vaccination), which can have a substantial impact on patterns of disease spread through the global population (22, 23).

Prevalence-based information includes direct factual knowledge about how commonly the disease occurs in a population (prevalence or incidence) as well as distribution of outcomes from a disease (e.g., number of cumulative deaths). For example, a previous study modeling human mobility patterns in response to an infectious disease epidemic assumed that people would avoid traveling to areas with a high disease incidence to minimize their risk of becoming infected (24). Belief-based information on the other hand includes information that influences people's beliefs and perceptions about the risks of disease, which may not have any correlation with the true disease situation. For example, individuals may choose to avoid vaccination because they perceive their risk of developing severe adverse vaccine reactions is greater than their risk of getting the disease even though this is statistically untrue (14). That is, prevalence-based information is based on *incidence*, whereas belief-based information is based on *incidents*. It is important to distinguish this difference when modeling dynamic human behavior because the former will fluctuate according to disease prevalence, whereas the latter has a more complex mechanism that operates largely independently from the disease dynamics.

Below, we first summarize the current literature about how prevalence-based and belief-based factors may influence farmers' decisions to either change the livestock contact patterns or adopt different control measures in response to a disease outbreak. We then highlight the key knowledge gaps that hinder modeling a dynamic human behavior.

### Prevalence-based factors

#### Local prevalence

There is evidence in the literature that suggests perception of risk—perceptions of threat, vulnerability, and severity—plays an important role in determining human health behavior (25, 26). Both human and livestock disease literature suggests that disease incidence influences perception of risk, which in turn affects uptake of disease preventive measures (13, 27). Local disease incidence, in particular, is often reported to trigger farmers' behavioral change. For instance, Garforth et al. reported that UK sheep farmers often demonstrated that they were willing to vaccinate animals against bluetongue once they heard the disease occurred in their region (28). Another qualitative study on Johne's disease suggested that farmers may not invest resources into controlling disease until they see clear evidence of disease on their farms (29). This “wait and see” attitude of farmers toward implementing on-farm biosecurity practices has been repeatedly reported in literature. For example, Alarcon et al. studied the reasons UK pig farmers decide to control disease and found that these reasons include observations of sick animals, reduced production, and increased mortality (30). Brennan et al. reported that some UK dairy farmers perceived that they can change the intensity of on-farm biosecurity practices when necessary, such as in the face of disease outbreaks (31). A rise in the local disease incidence can be, therefore, a legitimate parameter to model change in farmers' biosecurity practices, although there is a considerable knowledge gap on what threshold incidence may trigger behavioral change, or even whether or not such a threshold exists.

#### Global prevalence

The abovementioned UK sheep study also reported that a disease incidence at a wider spatial scale is less likely to motivate farmers to vaccine their animals against bluetongue (28). This relatively weak influence of global prevalence, as opposed to local prevalence, has been also reported for farmers' trading behaviors. Although some farmers, but not all, avoid purchasing livestock from a high disease risk area for both endemic (32, 33) and exotic diseases (34, 35), it is unclear whether or not farmers are engaged in the risk-averse trading in response to disease prevalence. In fact, a UK study showed that the proportion of farmers from low bTB risk areas who mentioned they do not purchase cattle from high risk areas was larger than that of farmers from high bTB risk areas; however, farmers listed maintaining an existing trade channel as the main reason for this behavior (36). Studies from New Zealand also suggested that the use of a stock agent may result in farmers' apparent risk-averse trading behaviors, although they may not be necessarily concerned about disease status (34, 35, 37). There was evidence that farmers avoid purchasing from certain geographical areas (34, 35); however, these areas may not necessarily have a higher disease prevalence than other areas or the area may represent a large geographical area (e.g., North Island of New Zealand rather than a specific region). Taken together, these may suggest that farmers' behavior is more likely to be influenced by their interpretation of disease prevalence, rather than the absolute prevalence, although this needs further empirical studies. This emphasizes the importance of belief-based factors, which we discuss below.

### Belief-based factors

The term “belief” is used to represent anything farmers believe; belief therefore includes perceived control of behavior (self-efficacy), perceived efficacy of behavior, perceived severity of disease, perceived benefit of controlling disease, social norm, and so on. In psychology literature, belief is assumed to form in one of three ways (38): (1) descriptive belief—personal beliefs arising from direct observations, (2) informational belief—beliefs arising from accepting information from outside, and (3) inferential belief—beliefs arising from processing other beliefs. When modeling human behavior and hence modeling belief formation, it is important to distinguish descriptive and informational belief: the former develops through farmers' personal experience and the latter through acquiring information from other actors such as peer farmers, veterinarians, government, and media. In this section, we focus on three key factors that contribute to a belief formation: disease experience, perception toward disease control measures, and social norm. We discuss how these factors develop both descriptive and informational beliefs.

#### Disease experience

One of the most studied factors that contribute to forming farmers' descriptive belief may be actual disease experience. Enticott et al. investigated practices and attitudes toward bTB among farmers in an area which had been recently designated as bTB endemic (32). This study found that the proportions of farmers that avoid purchasing from a high bTB risk area were similar between those previously had a bTB breakdown and those not (32), which may suggest a direct bTB experience may not necessarily change farmers' trading behaviors. On the other hand, a Dutch study suggested that previous direct experience of having bluetongue-related clinical cases was associated with a higher probability of vaccinating their livestock (39).

This discrepancy in the effect of actual disease experience on behavior change may be attributable to, at least in part, the difference in how risk perception is updated by the disease experience. Ferrer and Klein (26) summarized three types of risk perceptions recognized in health behavior discipline: (1) deliberative risk perception—systematic, logical, and rule-based perception to estimate, for instance, the likelihood of negative event occurring, (2) affective risk perception—affect associated with risk such as worry or anxiety about a threat of negative event, and (3) experiential risk perception—rapid judgements made from deliberative and affective perception, which can be described as intuition or “gut” feeling.

As has already been discussed, disease experience seems to contribute to high deliberative risk perception—after experiencing a disease, individuals may come to perceive that they are at high risk of contracting the disease. Studies also suggest that farmers who are not worried about future disease incidence (i.e., a low affective risk perception) have poor uptake of on-farm biosecurity practices (40). Interestingly, however, having both high deliberative and affective risk perception at the same time does not necessarily lead to implementing a preventive measure. For instance, a study investigating intention to quit smoking demonstrated that those who had high perception of risk—individuals that perceived they have a higher risk of contracting lung cancer—and high worry—individuals who were more anxious about contracting lung cancer—were more likely to have a lower intention to quit smoking (41). High levels of deliberative and affective risk perception may result in specific experiential perception which provides “fatalistic” belief about disease risk (26). Indeed, “fatalistic” belief has been observed among farmers who are at high risk of infection to disease which is difficult to prevent such as bTB (42) and Hendra virus (13). Farmers' emotion toward disease may be shaped by many events in their farming life and a single devastating event can also have a prolonged effect on their emotions. For instance, a study on UK farmers reported that a few farmers listed FMD, rather than other diseases, as a particular concern even though more than 4 years had passed since the 2001 FMD outbreak in the UK (43). These together emphasize the importance of understanding how previous experiences form farmers' emotion and how such emotion influences their behaviors, which is substantially missing in current literature.

Studies also suggest that peer farmers' disease experience is likely to act as informational belief. In fact, Lupton argues that risk perception and emotion are fluid, shared, and developed through interaction with others, material objects, and space (44). For instance, a study on UK pig farmers demonstrated that stories about negative impact of disease circulating among farmers triggered information seeking behavior of some individuals which did not have the disease (30). New Zealand farmers from a high bTB risk area also share a belief about bTB that they are always “one test away from being infected”—the local community developed understanding that bTB breakdown is unpredictable and inevitable after observing many bTB cases that occurred without any clear reasons (45). It is therefore important to model transmission of informational belief from affected farmers to other farms and how this updates individual's risk perception, and hence behavior.

#### Perception toward disease control measures

Beliefs including perceived efficacy and safety of control measures, perceived benefit of controlling disease, self-efficacy, and perceived behavioral control are similarly likely to transmit directly between farmers. Detailed descriptions for each term can be found in elsewhere (46, 47). Alarcon et al. reported that farmers may start implementing a specific disease control measure when they obtain “word of mouth” information on how effective the measure was on the other's farm (30). Studies also suggest that the lack of self-efficacy—one's belief to his/her ability to perform a behavior to obtain a desired outcome (48)—is shared by farmers within a community who observe disease control measures have not worked on other farms (42, 45). Wilson et al. argued that self-efficacy may be developed among a small group of farmers in which they share knowledge, experiences, and skills, which can lead to a behavioral change in the community (49).

These beliefs may also transmit between farmers indirectly through other actors such as veterinarians and farm advisors: farmers consider, at least to some extent, these actors to have good and reliable local knowledge (30, 46, 49). On the other hand, however, a longitudinal study of UK farmers suggests that farmers' views on disease control interventions may change little over time. This study tracked farmers' confidence in vaccinating badgers against bTB to help reduce disease in cattle (i.e., self-efficacy), farmers' confidence, and their trust in Government, identifying that these remained low throughout the duration of the study period (50). While disease prevalence appeared to be unrelated to vaccine confidence, the spread of stories of vaccine failures by local veterinarians and farmers were connected to declining confidence (51), suggesting veterinarians play a significant role in spreading information.

#### Social norm

Social norm has been frequently modeled in the context of human disease to account for human behavioral change, particularly for vaccine behaviors (52, 53). Social norm is often categorized into descriptive norm—perception about what is typically done—and injunctive norm—perception about what is typically approved and disapproved (54). Although within veterinary literature the influence of social norm on farmers' behavior has been repeatedly mentioned, there is only little knowledge on how social norm actually acts on farmers (55–57). As highlighted by Maye et al. (58), this lack of knowledge may arise from the lack of studies that separated the influence of social norm from attitude or the lack of studies that identified a full range of influential actors.

A study on farmer antibiotic use for mastitis treatment, however, provides interesting insight on how social norm influences the duration of antibiotic use by farmers (56). This study identified that the duration of antibiotic use was associated with the duration of clinical cure. However, the increased cost due to the extended antibiotic treatment (e.g., more waste milk) was not a concern for any of the farmers studied. The mastitis treatment practices of the studied farmers seemed to be little influenced by perception of society such as media and government, which tend to be against prolonged antibiotic use due to its potential association with the development of antimicrobial resistance. The authors hypothesize that farmers choose to provide the perceived best possible treatment, which farmers believe is approved to be a good practice by other farmers. Extended antibiotic treatment therefore provides farmers with a feeling of being a “good farmer” (56).

The concept of the “good farmer”—how the identity of being a good farmer influences farmer behavior—has been recently highlighted in social science studies. Naylor et al. identified three identities of “good farmer” in the context of exotic disease control (59); “good stockman”, “good neighboring farmer”, and “good public facing farmer”. Health and welfare of animals is valued by farmers with the “good stockman” identity, which may encourage farmers to identify and report suspicious disease quickly. Farmers with “good neighboring farmer” identity have a feeling of responsibility to local farmers, which encourages them to minimize disease spread to other farms. The last identity, “good public facing farmer,” is associated with maintaining a positive image of farmers' industry. The role of perception of responsibility on farmers' behavior has been similarly reported for (potential) zoonoses. A study on farmers' intention to control *Escherichia coli* O157 suggested that farmers who feel they are responsible for controlling the disease were more likely to be willing to use disease control measures (57).

As these studies highlight, pressure from peer farmers, industry, and society have, to some extent, an impact on farmers' behavior. However, there is currently a significant knowledge gap that prohibits modeling this impact. It has been recently shown that the influence from other actors on farmers' behavior varies depending on the context and disease. Using bTB as a case example, Maye et al. showed that while farmers perceived their decision to implement badger culling would be influenced by peer farmers, that for vaccinating cattle against bTB would be influenced by their veterinarians (58). This study result not only suggests the difficulty in determining which actors to include in modeling the impact of social norm on farmers' behavioral change but also raises an important question: Would farmers' decision to implement which control options be influenced by actors they perceive most important? In other words, farmers may simply decide to implement a practice recommended by a specific actor (e.g., veterinarian) because they perceive the actor's opinion important. Should this be the case, a question to ask is, who an influential actor is for farmers?—rather than, whether or not a specific actor's influence is important for farmer's intention to perform the practice. This is linked to the problem arising from looking at only single behavior, which we discuss in the next subsection.

### Knowledge gaps and limitations

#### Lack of understanding on qualitative behavior change of farmers

As highlighted above, there is relatively rich information on disease-related factors relevant to farmers' dynamic behavioral changes. Nevertheless, we have currently very limited knowledge on how these factors actually change their behaviors. This is partially due to the current research approach: A majority of studies focus on how economic and psychological determinants lead to a single behavior, which is pre-defined by researcher. This is reasonable if disease control is well-established and its option is very limited e.g., vaccination for exotic disease. Nevertheless, in reality, this is not the case for many important livestock diseases; farmers often have multiple options of disease control measures and it is unlikely farmers choose one measure through a full assessment such as a cost-benefit analysis (60, 61).

Indeed, recent studies suggested that each farmer develops a different control strategy depending on their situations, risk perceptions, and disease understanding (34, 35). If farmers are already implementing their “biosecurity” practices—which may be supported by previous findings that farmers feel they are doing sufficient practices—in response to disease and their farming experience, this raises an important question which is already covered by Shortall et al. (62): What does “good biosecurity” really mean to farmers and other actors? In the animal welfare context, it has been clearly highlighted that veterinary experts and farmers frame a behavior differently: while veterinary experts frame record-keeping practice is the key to improving animal welfare, farmers consider this practice as something to satisfy external accountability demands (63). This may well be the case for biosecurity practices—we assume a certain practice is essential to reduce disease risk, but farmers may have a totally different idea for the same purpose. This is a critical assumption we make, perhaps unconsciously. We need better understanding of why farmers choose a specific behavior—this is as important as why farmers do not practice a recommended practice, which is the focus of current literature.

As pointed out by Barnes et al., there is also a critical knowledge gap in the interactions between economic and socio-psychological factors on farmer decision making (5). This knowledge gap is critical not only for modeling farmers' behavior but also for improving overall biosecurity practice in livestock industry. Future studies are warranted to empirically and longitudinally observe how farmers actually change their behaviors (or not) in response to disease experience or disease outbreak and understand why they do so.

The literature also suggests that implementing disease preventive measures may reduce one's risk perception. This reduced risk-perception in turn changes one's other behaviors which are relevant to disease risk. This phenomenon is well-known as “risk compensation theory” (64). For instance, a study reported that horse owners relaxed horse and property management practices after they vaccinated horse against Hendra virus because their confidence in vaccination reduced the risk perception (13). Thus, biosecurity practices are interdependent on each other. Several studies provide useful information on the static interdependency between farm practices (65, 66), however, modeling farmers' behavior change requires knowledge on dynamic interdependency; how implementing one practice leads to a change in risk perception, and hence other behavior changes. An interesting insight is provided by a randomized control study on UK beef farmers. The authors assessed how tailored biosecurity advice may reduce the prevalence of selected diseases (67). This study found that farms in the intervention group that received specifically-tailored advice were significantly less likely to be seropositive for BVD and *Leptospira hardjo* in the end of the study period than those in the control group who received only generic advice. Nevertheless, farms in the intervention group were more likely to be positive for bTB in the end of the study period than those in the control group, despite the observation that biosecurity practices on farms in both groups were observed to be improved during this study. The authors speculated that farmers in the intervention group may have put more efforts to purchase from source farms that are accredited as free from diseases such as BVD and *L. hardjo*, which may not necessarily have been free from bTB for a long time (67). It is therefore important to understand whether or not farmers prioritize a specific disease over others and how this prioritization may change over time because a biosecurity intervention for or an experience of a specific disease can substantially change farmers' trading practice, which in turn may alter the infection risk to other diseases, as suggested by this study. Again, this knowledge gap can be only filled by investigating longitudinal changes in farmers' behaviors and perceptions in response to various disease-related factors—taking account of relationships between diseases rather than a single disease in isolation—and other wider factors such as animal welfare, environment, and economic components.

Many studies in the literature suggest that farmers will not continue to perform a practice if they do not perceive it to be effective, beneficial in terms of cost, or feasible in terms of both labor and cost (30, 46, 61, 68). Moreover, these farmers' assessments are not fixed in time—milk price, for instance, may drop and practices currently feasible may suddenly become costly. When modeling disease that spreads over a prolonged period of time or that can infect farms over multiple times, it becomes particularly important to account for the maintenance and cessation of changed behavior. Behavior change maintenance is, however, a neglected research area [but see (69) for a veterinary example and (70) for examples in human health].

#### Lack of understanding on the transmission mechanism of beliefs and information

A seminal study by Delabouglise et al. showed how information on poultry disease outbreak flows between stakeholders, and that this information is likely to trigger various farmers' behaviors such as implementing a preventive measure and selling animals (71). However, regarding transmissions of belief-based factors between farms, the literature provides inconsistent evidence. While agricultural studies suggest that information from trusted and credible farmers is the key determinant of one's uptake of knowledge and technology (72, 73), disease studies often highlight that farmers do not exchange their disease information (29, 30, 74). A lack of communication between farmers on disease problems may be attributable to stigma attached to disease and potential damage to farm's reputation (29, 56), which is particularly important if the farm sells animals to others (30). Disease information is likely to spread through specific social network of farmers and we need better understanding of the characteristics of such networks. For instance, important questions include: does such information network change between peace time and disease outbreak time? A longitudinal study on farmers' knowledge transfer, such as one by Wood et al. (75), can be carried out in livestock disease context and would provide useful information to fill this knowledge gap.

A belief-based factor transmission between farms via other actors, such as veterinarian, is another important pathway to be considered. Nevertheless, modeling this pathway is not straightforward for several reasons. First, the role of veterinarians on-farm disease prevention is still unclear. Shortall et al. reported that some veterinarians see their current role as “test and treat” rather than “predict and prevent” meaning that farmers often seek their advice only when they have problems (62). If informational belief relevant to disease prevention transmits from veterinarians to farmers after these farmers get infection (i.e., test and treat situation), for instance, modeling this belief transmission has a minimal impact on the change of farm susceptibility to disease infection—although such informational belief may have a large impact on within-herd disease transmission patterns. This, however, also means there is an interesting opportunity for modeling studies to demonstrate, for instance, how large the financial benefit that may be gained among farming communities, and countries, by shifting from “test and treat” to “predict and prevent” mode—this can be a good incentive for governments to invest onto a communication training for veterinarians so that they can be more involved in disease prevention.

Second, there seems to be large heterogeneity in veterinarians' advice and farmers' uptake of such advice. The former may be influenced by veterinarians' previous experience with specific measures (e.g., having positive or negative experiences with a specific vaccine), confidence in performing the intervention, knowledge of disease, and general attitudes toward disease (30, 62, 76, 77). The latter may be influenced by relationship and trust developed between farmers and veterinarians, and it is known that veterinarians often provide an advice and treatment tailored to each farmer (62, 78).

Third, little is known about how, why and to what extent veterinarians' practices, such as diagnosis and surveillance activity, evolve over time. This is particularly important for diseases such as bTB that are often non-detectable by farmers—veterinarians define a farmer's disease experience. Enticott identified that bTB surveillance protocols employed by veterinarians are adapted to the situation at hand: Shortcuts are learned and passed on between veterinarians within veterinary practices in doing so developing their own cultures of testing, both creating and reflecting what are seen to be the central facets of the “good vet” and veterinary identity (79). Studies have, therefore, shown variation in performance between veterinarians in areas of different disease prevalence (80), and where organizational structures and cultural distance between veterinarians and farmers varies (81). For example, vets working for government organizations find more disease than those in private practice who test their own clients' cattle (82), while other studies find differences in performance between male and female vets (83). These variations in behavior are not strictly confined to veterinarians either; studies of the detection of disease at post-mortem have revealed significant differences between abattoirs (84). While these variations may call into question apparent objectivity of disease data, they also suggest the need for greater understanding—both of why variations occur (and what can be done about it), and whether these behaviors change over time in relation to the spread of disease.

These, together, emphasize the need for better understanding of how information spreads between farmers and other actors and how this might change farmers' behaviors. Further studies are warranted in order to incorporate these mechanisms into a disease simulation framework without them being too complex.

## Methods for modeling dynamic human behavioral changes

This section focuses on reviewing the methods used to estimate quantitative associations, which are the information required to incorporate a dynamic behavior into a disease simulation model. Theoretical studies often model these associations using either of two major approaches—economic or psychological models. We briefly highlight previous applications of these two approaches. Then limitations of these approaches are discussed and we highlight other potential approaches to associating a human behavior to a disease-related factor. More details of these methods as well as applications to human diseases can be found in recent excellent review papers (6, 7).

### Economic models

For livestock diseases, the seminal papers in this field exclusively model human behavior in an economic framework: that is, a disease-related factor is an economic cost incurred by a disease and a behavioral change occurs to minimize such a cost. In particular, game theory has often been applied to understand an interdependent nature of decision making on infectious disease control. It assumes that one's decisions about controlling an infectious disease influences local disease epidemiology and hence the disease risk imposed on others, which in turn influences others' decision making. Typically, these studies focus on one particular behavior, either a single biosecurity practice or a single trading practice. For instance, Hennessy modeled farmers' on-farm biosecurity practices using a simple spatially structured disease model, which accounted only for farm profit (85). Kobayashi and Melkonyan performed a theoretical and empirical study using farmers' biosecurity behaviors at a livestock show in California to investigate how the decisions made by individual farmers in a trading pair influenced each other's subsequent decisions (86). Murray applied game theory to an aquaculture setting to model whether or not fish farmers purchase tested pathogen-free stock or untested stock that may carry a pathogen (87). This study identified that the key motivator for a farmer to uptake a disease preventive measure is often the confidence in other farmers performing the measure.

Given that the objective of these studies is to identify a disease control strategy that maximizes the collective benefit under a given human behavior, they typically use very simple disease transmission models. An exception is the work by Tago et al., which modeled dynamic livestock selling behavior of farmers in an economic framework and simulated disease spread using both a network-based model and a spatial transmission model (88). This study showed how an inferred effectiveness of a movement restriction policy on a disease spread is overestimated when a dynamic behavioral response is ignored. Hoscheit et al. modeled French livestock movement patterns accounting for livestock supply and demand, although disease-related factors were not considered in this study (89).

These studies however typically consider only a one-off behavioral change. As an exception, Rat-Aspert and Fourichon modeled a dynamic voluntary vaccination behavior that changes according to a disease prevalence, which in turn influences an economic incentive of vaccination (90). However, the behavioral change in this study is assumed to occur only once a year and farmers' decision to vaccinate does not get updated in response to a disease spread situation.

### Psychological models

The other class of approaches to modeling human behaviors use psychological models. Unlike economic models, psychological models do not make the assumption that humans behave in a manner to maximize a certain utility. Rather, they assume various psychological factors have an independent association with an intention to perform a certain behavior, which in turn associates with the actual performance of a behavior. The psychological factors used to model a behavior depend on different models. For instance, Theory of Reasoned Action (TORA) assumes one's intention to perform a specific behavior can be explained by one's attitude and subjective norm toward the behavior. One's attitude is in turn determined by a belief about, and evaluation of the outcomes of the behavior (91). Theory of Planned Behavior (TPB), an extension of TORA and a popular approach in recent veterinary epidemiology literature, assumes that one's perceived behavioral control toward the behavior also influences one's intention, in addition to the two factors in TORA. This additional component of TPB implicitly accounts for self-efficacy, which is one's belief that one can achieve the behavior, and other factors facilitate achieving the behavior such as personal skills, information, opportunities (92). The Health Behavior Model (HBM) has also been frequently applied to human diseases, but less so for livestock diseases (93). This model assumes engagement in a specific behavior toward a disease can be explained by factors such as one's belief about the disease problem, perceived benefits of a behavior, and self-efficacy of a behavior (94). The greatest strength of these models is that the probability of performing a specific behavior can be quantitatively described by these factors using a questionnaire survey. These approaches have been often used to investigate why farmers do and do not engage in a specific biosecurity behavior.

However, one notable exception is a recent work by Fischer et al. (95). Using an individual-based model framework, this study accounted for farmers' dynamic treatment behaviors with antibiotics, which in turn influence how disease spreads within a farm. Farmers' dynamic behavioral change was modeled using TPB and their intention to change behaviors is assumed to depend on three factors; the expected economic gain from changing a behavior, the satisfaction in their own behaviors, and social norms. Although this model still includes several strong assumptions (e.g., farmers have perfect information regarding the cost of measures and the actual behavior is determined by an intention to perform the behavior), it is an excellent example of incorporating dynamic human behaviors in a disease simulation model.

### Knowledge gaps and limitations

Traditional economic frameworks often assume that humans behave in a manner such that it maximizes a certain utility. Game theory assumes that individuals have perfect information as to the cost and effectiveness of a disease control strategy. It is, however, increasingly known that human behaviors do not hold to these assumptions. In reality, farmers do not have sufficient information to evaluate the true cost and effectiveness of a control measure. Moreover, as we discuss in the next section, it is unlikely that farmers go through a full cost-benefit analysis on a control measure accounting for the influence from others' decisions.

Although the ability to quantify an association between each psychological factor and a resulting behavior is beneficial, particularly for modeling studies, these methods are not without limitations. First of all, TORA and TPB were not originally developed to model behavioral changes (96); although they have been applied for this purpose in many studies, the validity of modeling behavioral change using these methods remains unclear. Second, there is evidence of a discrepancy between intention and actual behavior (97, 98), the so called intention-behavior gap, which fundamentally violates the assumptions of these models. In fact, it has been long recognized that having an intention to perform a behavior is often insufficient motivation to actually carry out that behavior (99). Third, these models do not explicitly account for how experience of performing a certain behavior influences cognitions, which are the impacts of doing the behavior on a person's attitude, subjective norm, and perceived control (100). Literature on farmers' adoption of new technology suggests that establishment of new practices takes time, going through an active assessment period, an implementation period, and a consolidation period where farmers iteratively seek options, invest resources to implement the new practice, and evaluate its effectiveness (101). Therefore, the lack of a mechanism that captures the process of establishing new behavior may be a constraint in modeling farmer behavior, as it is known that farmers are more likely to implement practices that they are experienced in performing (61).

Not relying on these models, Higgins et al. investigated how veterinary clinicians make a treatment decision based on a result from the previous treatment action (77). The authors compared observed clinicians' treatment practices to those theoretically predicted assuming they logically update their beliefs using a Bayesian framework. Although farmers' treatment decision may not exactly match to that of clinicians, the decision of clinicians should be still influential to farmers' decisions given farmer reliance on veterinarians to advise about the best course of action for disease issues (28). This study provides useful information for the disparity between actual human behaviors and expected behaviors that are derived from a certain theory.

While there are no applications in the context of livestock diseases, diverse theories have been developed, tested for their validity, and used for modeling behavioral change in other disciplines. Several key distinctive features of these theories include acknowledging: non-conscious factors (e.g., impulsive and automatic factors) (102), cognitive habits and socially shared values (103), and emotions (104, 105). Theoretical models are useful in that they can readily inform researchers of factors they may want to consider when investigating a specific behavior. Nevertheless, with a significant difference between a health behavior and on-farm behavior, we contend that we may need to develop a tailored behavioral change model in this field rather than borrowing models that are developed for other purposes. This can only be achieved through acquiring more knowledge of farmers' behaviors and their behavioral changes using empirical qualitative and quantitative studies.

## Discussion

Throughout this manuscript, we have highlighted knowledge gaps and limitations specific to two questions that need to be answered to model dynamic human behavioral changes: (1) the disease-related factors that are most relevant to motivate behavioral change, and (2) the quantitative association between a change in these disease-related factors and a change in a behavior. Here, we list six general challenges in veterinary epidemiology that we need to overcome to improve our understanding of human behavior.

### Challenge 1: little focus on capturing farmers' true behaviors

Many current studies on farmer behavior rely on questionnaire survey asking self-reported practice; however, this type of study needs a careful consideration because the discrepancy between self-reported and actual behavior has been repeatedly identified (106–108). It may be the time to employ a more rigorous qualitative method such as biographical narrative interpretive method (29, 109)—a method to acquire interviewee's real-life experience—and quantitative studies using objective measures of farmers' behaviors (67, 110, 111).

### Challenge 2: lack of empirical longitudinal data

We contend that employing theoretical psychology models to predict behavior may be a useful quantitative tool but the validity of and assumptions behind models should be rigorously examined rather than merely applying a model to data (112). These models identify only “correlations” between psychological factors and a causal model for behavioral change remains unknown (113, 114). We need more longitudinal studies that follow how actually farmers' attitude, perception, belief and behavior change over time in response to various factors; not only disease-related but also wider animal welfare, environment, and economic factors because these can all lead to a change in farm biosecurity practices although improving biosecurity may not be farmers' primary purpose. There is much to learn from human health behavior discipline, where various interventions to change human behaviors have delivered a mixture of success and failure (115). Analysis of increasingly available big data is also useful to validate findings from in-depth qualitative studies and provide a hypothesis on human behavior patterns, which can be further investigated in qualitative studies. Such big-data analysis itself should be carried out by accounting for miscellaneous bias arising from human behaviors—data is essentially a product of, for instance, a decision to participate in a surveillance system and report a disease case (116).

### Challenge 3: tendency to focus on a single disease

Another significant challenge is the development of a disease model that captures dynamics of multiple infectious diseases. Most available models simulate a single disease spread. However, spread patterns of each disease is not independent. As highlighted in this review, disease spread influences farmers' behaviors and trading patterns, which in turn will influence the spread of other infectious diseases. Modeling multiple diseases can be complex and computationally expensive: nevertheless, we do not need to simulate every single disease because humans cannot make a decision considering many complex factors (e.g., diseases) either. We need to understand farmers' decision making from their perspective.

### Challenge 4: barriers to interdisciplinary collaboration

Of course, the call for greater interdisciplinary working has been made by others working in the field of animal disease (117, 118). Nevertheless, institutional boundaries and disciplinary norms can frustrate good intentions (119), rendering interdisciplinarity an attractive but distant prospect. Potentially, as suggested in this review, a focus on the dynamic nature of human behavior may provide both disciplinary and interdisciplinary methodological and theoretical challenges, in doing so creating a critical mass that overcomes barriers to interdisciplinary working.

### Challenge 5: gaps in framing behaviors between scientists and lay people

As highlighted in this review, the fundamental problem may be that we try to answer why farmers do not practice a certain behavior, which we pre-defined. Farmers, however, frame behaviors differently from we do. What if farmers are aware of disease problem but implement their own “biosecurity” practices they believe effective? This review clearly points out, from behavior modeling perspective, that we lack understanding of “how” farmers change their behaviors.

### Challenge 6: over-simplification vs. over-modeling

As highlighted throughout this manuscript, the dynamics of human behavior can be challenging to model, especially when there is significant heterogeneity in behaviors between different groups of farmers. One may therefore argue that these complexities can be ignored as long as the model inferences are robust to sensitivity analysis. However, it should be noted that the most commonly used sensitivity analysis in veterinary epidemiology evaluates only the impact of parameter uncertainty and not the uncertainty in the model structure itself (120). Whether or not a specific dynamic behavioral component needs to be considered can be only evaluated by comparing inferences from models with and without the component, and this evaluation may be necessary for different diseases, populations, time-scales, and objectives of the study (3, 8, 121). While we contend unnecessary complexities should be avoided, it is important to carefully evaluate if the simplicity of a given model adequately fits for the study objective (122, 123).

## Conclusion

An existing collaborative environment between scientists from veterinary epidemiology, animal welfare, and social science provides an exciting opportunity to provide a better understanding on behaviors and decision making of not only farmers, but also humans in general. At the same time, within the discipline of epidemiology itself, more theoretical studies that incorporate dynamic human behavior and detailed infectious disease modeling continue to be necessary to identify behaviors that we should focus on understanding more. Studies should be self-critical about making unconscious and conscious assumptions—be it a behavioral study based on existing theories or a modeling study for an infectious disease spread—and discuss potential biases inherent to making such an assumption.

## Author contributions

AH designed and drafted the paper. GE contributed to the important content in the draft and critically revised the paper. RC and MG critically revised the paper and contributed to important intellectual content.

### Conflict of interest statement

The authors declare that the research was conducted in the absence of any commercial or financial relationships that could be construed as a potential conflict of interest.
